# Development of Siglec-9 Blocking Antibody to Enhance Anti-Tumor Immunity

**DOI:** 10.3389/fonc.2021.778989

**Published:** 2021-11-19

**Authors:** Hyeree Choi, Michelle Ho, Opeyemi S. Adeniji, Leila Giron, Devivasha Bordoloi, Abhijeet J. Kulkarni, Alfredo Perales Puchalt, Mohamed Abdel-Mohsen, Kar Muthumani

**Affiliations:** Vaccine & Immunotherapy Center, The Wistar Institute, Philadelphia, PA, United States

**Keywords:** human Siglec 9, NK cell, monoclonal antibodies, ovarian cancer, immunotherapy

## Abstract

Sialic acid-binding Immunoglobulin-like lectin-9 (Siglec-9) is a glyco-immune negative checkpoint expressed on several immune cells. Siglec-9 exerts its inhibitory effects by binding to sialoglycan ligands expressed on cancer cells, enabling them to evade immunosurveillance. We developed a panel of human anti-Siglec-9 hybridoma clones by immunizing mice with Siglec-9-encoding DNA and Siglec-9 protein. The lead antibodies, with high specificity and functionality against Siglec-9, were identified through screening of clones. The *in vitro* cytotoxicity assays showed that our lead antibody enhances anti-tumor immune activity. Further, *in vivo* testing utilizing ovarian cancer humanized mouse model showed a drastic reduction in tumor volume. Together, we developed novel antibodies that augment anti-tumor immunity through interference with Siglec-9-mediated immunosuppression.

## Introduction

Immune checkpoints are conventionally responsible for preventing unregulated immune responses to ensure limited collateral damage to surrounding cells (1–5). However, cancer cells can engage these immune checkpoints on immune cells to render them inactive/anergic, ensuring self-survival and growth (4). Thus, one important approach of cancer immunotherapy is the usage of immune checkpoint inhibitors [including monoclonal antibodies (mAbs)] with the ability to block the immuno-regulatory interactions between tumor and immune cells (4, 6, 7).

One emerging class of immune checkpoints is Sialic acid-binding Immunoglobulin-like lectin (Siglec) receptors, which are single-pass transmembrane I-type lectins present on hematopoietic cells (5, 8–10). Siglecs are key immunomodulatory receptors expressed by several types of immune cells, such as eosinophils, neutrophils, macrophages, natural killer (NK) cells, dendritic cells, B cells, and T lymphocytes (11). Siglecs facilitate activation and inhibition of immune responses through immunoreceptor tyrosine-based inhibitory motifs by interactions with sialogylcans on cancer cells (5, 11). The expression of sialoglycans on tumor cells surfaces facilitates tumor survival as well as growth by preventing recognition during immunosurveillance (12, 13).

Among Siglecs, Siglec-9 is expressed on myeloid cells, NK cells, and a subset of T cells (7, 14–23) and can bind to sialoglycans (on cancer cells), resulting in inhibiting anti-tumor immune responses (7, 17, 24). For instance, Siglec-9 expression on NK cells can inhibit anti-tumor immunity (17). Siglec-9 is also expressed on a subset of CD8+ T cells in the tumor microenvironment (7), and its interactions with sialic acid inhibit CD8+ T cell functionality (7). Finally, Siglec-E (the mice homolog of Siglec-9) on mice neutrophils and tumor-associated macrophages promotes cancer cell metastasis, induces apoptosis of neutrophils, and aids in the formation of a pro-tumorigenic phenotype of macrophages (25).

Owing to the important immunomodulatory roles of Siglec-9 in the tumor microenvironment, it has gained attention as a target to achieve enhanced anti-tumor immunity. Siglec-sialic acid interactions are important immune negative checkpoints against autoimmunity (26–29), and several Siglec members share high homology (9). Therefore, the success of targeting Siglec-9 as a potential immunotherapy approach hinges on the availability of highly specific antibodies to Siglec-9. In this study, we generated and characterized monoclonal blocking antibodies against human Siglec-9 with anti-tumor activities *in vitro* and *in vivo*.

## Results

### Generation, Expansion, and Binding Characterization of Human Siglec-9 mAbs

**Figure 1:**
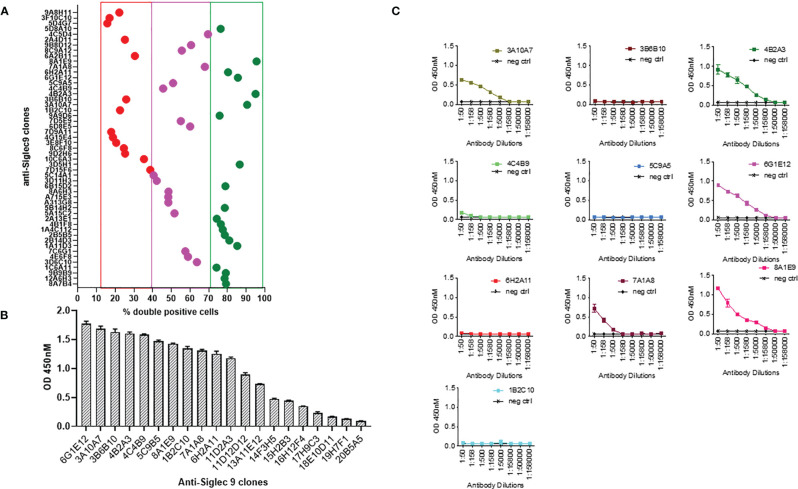
Generation of monoclonal antibodies against human Siglec-9. **(A)** Mice were first immunized with human Siglec-9 DNA (2x), then boosted with recombinant human Siglec-9 protein to generate a humoral immune response and tested for antibody levels. Spleens from the mice were harvested, fused with 2P2/0 mouse Myeloma cells. Developed hybridomas were exhibiting anti-human Siglec-9 expression confirmed by ELISA and IntelliCyt FACS assay. Analysis of top 50 candidates: flow cytometry-based screening to evaluate anti-human Siglec-9 binding; Siglec-9 overexpressing K562 cells were first stained with hybridoma supernatants primary test and subsequently with APC-conjugated anti-mouse secondary antibodies followed by analysis using Intellicyt iQue screener PLUS system. GFP+ and APC+ cells were gated using IntelliCyt iQue screened PLUS system, where high % double-positive cells were identified. **(B)** Binding ELISA of top 20 GFP+ and APC+ %double positive anti-human Siglec-9 mAb candidates by ELISA. Recombinant human Siglec-9 protein was used in ELISA to assess the antigen-specific binding. **(C)** Screening and titration of serially diluted mAb candidates. Recombinant human Siglec-9 protein was used as a coating antigen for binding ELISA. The positive control shows responses, and the negative control (recombinant HIV-1 gp120 protein) shows no response with anti-human Siglec 9 hybridomas. Each point represents the OD value with the mean ± standard deviation from three determinations.

Mice were immunized with human Siglec-9 to generate a humoral immune response. Spleen from the immunized mice was harvested and used for the development of hybridomas expressing anti-human Siglec-9 antibodies. Following the intramuscular injection of mice with an expression vector containing DNA for human Siglec-9, a strong humoral immune response was noted, and antibodies to human Siglec-9 were observed in the sera of all mice. The antibody reaction became more substantial, and the polyclonal antibody titer against human Siglec-9 remained high after subsequent injections. Hybridomas were incubated for 2-3 weeks after fusion with 2P2/0 mouse myeloma cells. A total of 1152 mAb clones (hybridoma supernatants) were screened for anti-human Siglec-9 binding activity by ELISA (data not shown). An additional flow cytometry-based screening test called IntelliCyt FACS analysis was performed on the top 50 candidates from the ELISA screening. Briefly, Siglec-9-GFP overexpressing K562 cells were stained with primary test hybridoma supernatants followed by secondary APC-conjugated anti-mouse antibodies and analyzed by IntelliCyt iQue screener PLUS based FACS analysis. Gating was done for double-positive cells (GFP^+^ and APC^+^ staining) and top 20 candidates that conferred high hits, 17 intermediate from Intellicyte screenings high % double positive hybridoma clones (**Figure 1A**). We again performed an ELISA assay for the top 20 anti-Siglec 9 mAbs from the IntelliCyt iQue screener PLUS-based FACS analysis at 1:50 dilution to further confirmed binding (strong binding = OD>0.6 at 450nM) (**Figure 1B**). **Figure 1C** shows tertiary hybridoma screening for the best ten clones, which were analyzed by serial dilutions of hybridoma supernatant by ELISA with recombinant human Siglec 9 protein. We included negative and positive control wells to confirm the results of primary and secondary screening and rule out the cross-reactivity and specificity. A high level of binding was achieved for the 8A1E9 clone, and we expanded further characterization of this clone. Based on this analysis of high binding capacity to human siglec-9, the selected 8A1E9 clone was moved onto the large-scale amplification/expansion for further characterization.

### Recombinant Antibody Expression in HEK293 Cells and Functional Characterization

**Figure 2:**
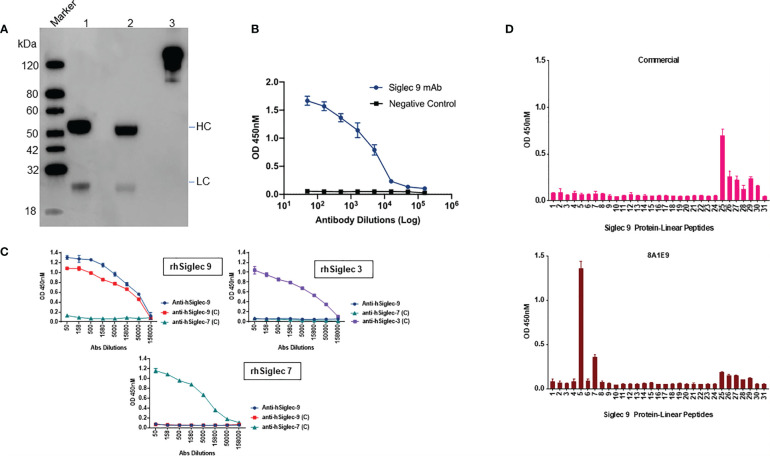
Characterization of recombinantly expressed human Siglec-9. **(A)** Western blot analysis of recombinantly expressed anti-Siglec-9. Recombinant antibodies are expressed monoclonal antibodies that are generated *in vitro* using synthetic genes. The purified recombinant antibody was analyzed by SDS-PAGE, Western blot analysis to determine the molecular weight and purity under non-reducing (lane 3) and reducing conditions (lane 2) alongside isotype control human IgG1, Kappa antibody (lane 1). Reducing and non-reducing loading buffer was added to protein sample respectively, and the final concentration of protein was 0.5 mg/ml. HC, Heavy Chain; LC, Light Chain. **(B)** Siglec-9 antigen binding of recombinantly expressed Siglec-9 antibodies was measured by ELISA. Assay plates were coated with recombinant Siglec-9, and recombinantly expressed mAbs were used. The binding was determined by ELISA. **(C)** Recombinantly expressed monoclonal antibodies were tested by ELISA to determine the binding specificity of anti-hSiglec-9. Assay plates were coated with recombinant human Siglec 3, 7, and 9 and then probed with recombinantly expressed anti-hSiglec-9 as well commercial antibodies (labeled as C) against each other to test immune cross reactivity. Anti-hSiglec-9 showed binding specificity to only recombinant hSiglec-9 and did not exhibit any binding to hSiglec-3 or hSiglec-7. **(D)** To determine potential epitope(s) regions for anti-Siglec-9 antibody binding, an indirect ELISA was performed. Peptide-based ELISA was performed using 100 μL/well of peptide at 2 μg/mL. ELISA plates were coated with 20mer peptides of human Siglec 9 protein as indicated, and recombinant anti-Siglec 9 and commercial antibodies samples were diluted 1:50 and analysed by ELISA as indicated in the Materials and Methods. Data are mean ± SDs for three wells (representative of two independent experiments).

We adopted the HEK293 mammalian cell line grown at high densities in suspension and was used for recombinant antibody expression. The antibody cloning method represents a simple two-step protocol with complete design flexibility. Antibody sequence (VH and VL) encoding clone 8A1E9 nucleotide sequences were optimized for humans and then was cloned into optimized human IgG1 and then constructed in pCDNA3.4 expression vector for recombinant production. The purity and apparent molecular weight of purified antibodies were assessed by SDS-PAGE analysis. The molecular sizes corresponding to the heavy chains (HC) (50-60 kDa) and light chains (LC) (25 kDa) suggested that the secreted antibodies are properly folded (**Figure 2A**). **Figure 2B** depicts recombinant expressed antibody (clone-8A1E9) binding to recombinant human Siglec-9 protein as measured by ELISA. Further, ELISA analysis was carried out to determine the specificity of recombinant anti-Siglec-9 against recombinant human Siglec-3, Siglec-7, and Siglec-9 antigens. Anti-Siglec 9 only exhibited significant binding and showed specificity to recombinant human Siglec-9 protein, whereas no binding was detected with recombinant Siglec-3 or Siglec-7 proteins (**Figure 2C**).

Next, we assessed the functional characteristics of the recombinantly expressed anti-Siglec-9 (8A1E9) antibody. Indirect ELISA was used to determine potential epitope(s) for anti-Siglec-9 antibody binding. In this assay, 20-mer peptides were generated against human Siglec 9 protein, and commercially available anti-Siglec-9 antibody was used as antigens to identify the epitope. Data from the analysis show that the recombinantly expressed antibody (clone-8A1E9) binds strongly to peptide # 5 and moderately to peptide # 7 (**Figure 2D**). No overlap between epitopes for the antibody (clone-8A1E9) and commercial anti-human Siglec-9 antibody was observed.

### Anti-Siglec-9 Antibody Enhances Human NK Degranulation

**Figure 3:**
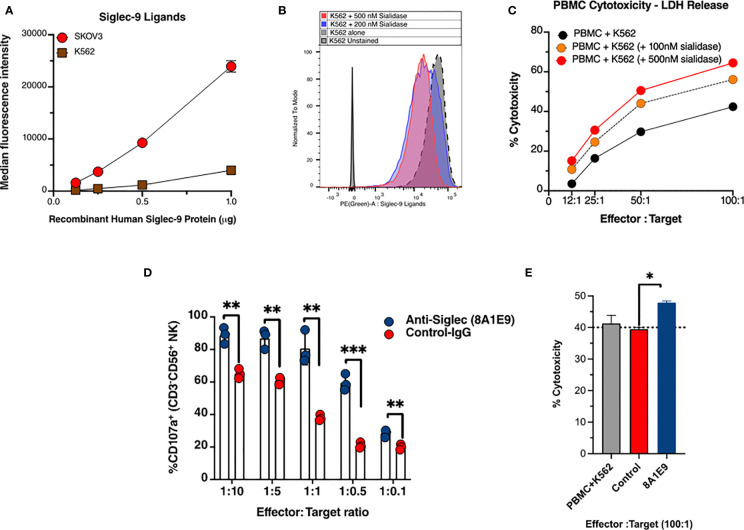
Anti-Siglec-9 mAb is functional and enhances anti-tumor immune activities *in vitro*. **(A)** Cell surface Siglec-9 ligand expression on K562 and SKOV3 cells. An equal number of indicated cells were incubated with varying amounts of recombinant human Siglec-9 Fc protein. The binding of Siglec-9-Fc to cells was measured using PE anti-human Fc fluorescent secondary antibody. **(B)** K562 cells were treated with 200nM or 500nM sialidase for 1 h at 37˚C and then were incubated with 1µg recombinant human Siglec-9 Fc protein. The binding of Siglec-9 Fc to cells was revealed using PE anti-human Fc fluorescent secondary antibody. **(C)** K562 (desialylated or not) cells were co-incubated with PBMCs from a healthy donor at different effector to target ratios. Cytotoxicity was determined by the LDH assay. **(D)** Measurement of NK cells activity against K562 target tumor cells with and with anti-Siglec-9 recombinant mAb (8A1E9 clone). Effectors are NK cells fixed at 200,000 cells. **(E)** Evaluation of antibody-dependent cell cytotoxicity using lactate dehydrogenase (LDH) measurement. The cytolytic activity of human Peripheral Blood Monolayer Cells (PBMCs) against K562 (NK-sensitive tumor cells) targets in the presence of anti-siglec-9 antibodies (1:10 dilution). Cytotoxicity was determined by measuring the amount of endogenous lactate dehydrogenase (LDH) released into the media. The assay was performed at ~100:1 effector-to-target ratio per well; triplicate. PBMC+K562 is the baseline cytotoxicity with no antibody. * = p<0.05, ** = p<0.01, and *** = p<0.001.

To evaluate the effects of the anti-Siglec-9 (clone -8A1E9) on NK cell degranulation, Siglec-9 ligands expression on the tumor cell lines were used in the study was tested to mitigate the validity of the specificity of anti-Siglec-9 mAb generated. Therefore, cell surface Siglec-9 ligand expression was characterized on ovarian cancer cell line SKOV3 and human leukemia cell line K562 cells. SKOV3 or K562 cells were incubated with varying amounts of recombinant human Siglec-9 Fc protein. The binding of Siglec-9 Fc to cells was measured using an anti-human Fc fluorescent secondary antibody (**Figure 3A**). Removing these ligands using sialidase (**Figure 3B**) enhances PBMCs-mediated Cytotoxicity against these cells (**Figure 3C**).

We then examined the effects of anti-Siglec 9 antibody (8A1E9 clone) on NK cytotoxicity against K562 cells. K562 cells were incubated with primary NK cells in the presence of anti-Siglec 9 antibody or control IgG. Following incubation, NK cells were stained with antibodies against CD16, CD56, and CD107a and evaluated by flow cytometry. The expression of CD107a corresponds to the degree of degranulation of NK cells. The plot of % CD107a^+^ *vs*. effector: target (NK cells: K562 tumor cells ratio, where NK cells were fixed at 200,000 cells, shows that the anti-Siglec 9 antibody (8A1E9 clone) induced higher degranulation of NK cells compared to control IgG (**Figure 3D**).

### Anti-Siglec-9 Monoclonal Antibody Enhances Human PBMCs Toxicity Towards Cancer Cells

To test for the 8A1E9 antibody’s ability to enhance immune cytotoxicity, PBMCs were co-cultured with K562 cells, with and without anti-Siglec-9 antibodies (at 100:1 effector: target ratio). In the presence of anti-Siglec-9 antibody, % cytotoxicity was increased, as measured by the amount of LDH released from these cells (normalized to the background as described in the methods section), suggesting the involvement of Siglec-9 antibodies in the restoration of immune functions against cancer cells (**Figure 3E**).

### Anti-Siglec-9 Antibody Reduces Tumor Volume in a Humanized Mouse Model of Ovarian Cancer

**Figure 4:**
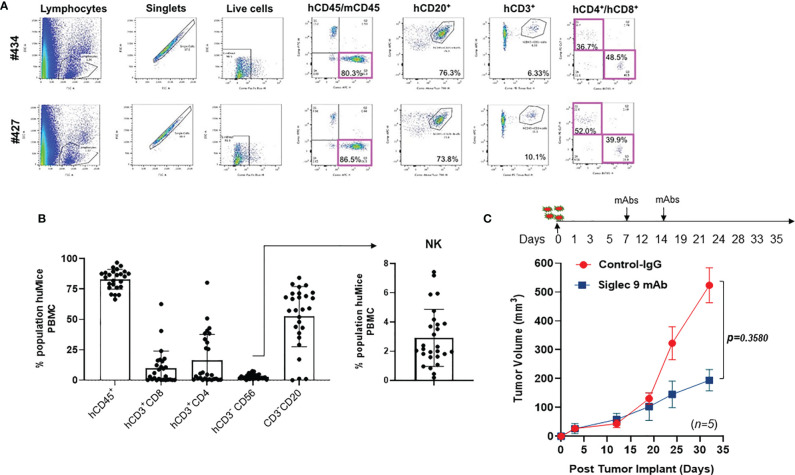
Anti-Siglec-9 mAb reduces tumor volume *in vivo* in a humanized mouse model of ovarian cancer. **(A)** Dot plots showing selected windows and gating strategy as applied to the identification of major immune cell populations in the hu-mice. Percentage represents the means of values obtained in 2 independent experiments. **(B)** Representative flow cytometry histograms of FACS analysis immune cell populations in the hu-Mice. Each marker was tested on at least three independent experiments and created from a batch of 27 mice; hCD45+ show n=25 dots, CD20 show n=27 dots, CD56 show n=27 dots. Data were calculated as average % of expressing cell ± SD. All panels are representative of 3 independent experiments. **(C)** Effects of anti-human Siglec-9 antibody on tumor growth in hu-mice. On day 0, humanized mice (n=5) were injected with 5x105 SKOV3 cells. 100µg of anti-hSiglec-9 antibody or control IgG was administered to each mouse on days 7 and 14. Tumor volume was measured on days 3, 12, 19, 24, and 33. After tumors became detectable, tumor masses were measured with a manual caliper, and tumor volumes were calculated, approximating the tumor mass to a sphere, according to the following equation: tumor volume = ½ (length x width2).

Wild-type mice do not express human immune cells. Hu-mice is a humanized mouse model that harbors functional human immune cells that respond to tumor challenges (30). Hu-mice were reconstituted from immunodeficient NSG (NOD.Cg-Prkdcscid IL2rgtm1 Wjl/SzJ) mice with HLA-A allele matched CD34^+^ hematopoietic cells and thymic cells (placed under renal capsule) and customized cytokine cocktails delivered by DNA (30). The reconstituted hu-mice showed strong repopulation of a diverse human immune repertoire (**Figures 4A, B**). To test *in vivo* the efficacy of the anti-Siglec-9 8A1E9 antibody, we implanted SKOV3 ovarian cancer cells (a cell line expressing high levels of Siglec-9 ligands; **Figure 3A**) subcutaneously in 2 groups of hu-mice. Seven days post tumor implantation; we treated one group with 100µg of 8A1E9 antibody and the other group with control antibody on days 7 and 14. Siglec-9 blockade by 8A1E9 was able to significantly reduce tumor burden in this hu-mice (**Figure 4C**).

## Discussion

Siglec-9 (expressed on the surface of several immune cells) can bind to its sialic acid-containing ligands (overexpressed on cancer cells). This binding exerts a negative signaling cascade that eventually inhibits the functions of these immune cells. Notably, the Siglec-9-mediated inhibition of immune functions is MHC-independent; therefore, cancer cells can utilize this mechanism to evade host immune surveillance (31). Indeed, emerging evidence suggests that Siglec-9 is an important glyco-immune negative checkpoint that can be targeted to enhance immune functions against cancer and virally infected cells (5, 20, 22, 24). However, the success of such an approach hinges on the availability of specific monoclonal antibodies that can block Siglec-9 and induce immune functions. In this short report, we developed such an efficient antibody and tested it *in vitro* and *in vivo*.

Siglec-9 shares around 84% sequence homology with Siglec-7 (21). However, our lead antibody showed no binding to Siglec-7 (or to Siglec-3) and a high binding to Siglec-9. This data suggest that our novel antibody can be used, in the future, to selectively target Siglec-9 without impacting the functions of other Siglecs. This is important as many of these Siglec interactions are important against autoimmunity (26–29). This high selectivity of our lead antibody was coupled with its ability to significantly enhance immune functions against cancer cells *in vitro* as well as to reduce ovarian cancer progression *in vivo* in a humanized mouse model. Together, these data suggest that this antibody represents a promising opportunity to target Siglec-9 interactions in a selective and efficient manner.

Siglec-9 is expressed on myeloid cells, NK cells, and a subset of T cells (7, 14–23). It will be important, in future studies, to examine the contribution of each of these cell types in our observed *in vivo* anti-tumor effects. This can be achieved by depleting each of these cell types before the cancer challenge. Also, it will be important to determine the potentially additive effect of combining these antibodies with other cancer therapeutic approaches. Finally, in addition to Ovarian cancer, Siglec-9 interactions have been described to be important for the progression of Melanoma (7) and Pancreatic cancer (20). Beyond cancer, these interactions have also been shown to play a role in modulating viral infections (22). Testing our lead antibodies in other cancer models and models of infectious diseases should be the subject of future studies.

In summary, we used a novel approach to create a DNA plasmid that encodes the Siglec-9 antibody sequence with the codon and mRNA optimized for improved selectivity and *in vivo* expression. Immunological and molecular characterization assays were used to down select clones with the highest affinity for Siglec-9 using recombinant Siglec-9 protein. Binding ELISAs and Western blot showed Siglec-9 mAb clone to bind to only Siglec-9 protein. We have also tested our Siglec-9 antibody’s ability to enhance anti-tumor functions *in vitro* and its ability to generate anti-tumor immunity in tumor-bearing mice. Indeed, anti-Siglec-9 antibodies reduced tumor burden *in vivo*. The findings observed from this investigation support the importance of this novel Siglec-9 immune-checkpoint and the potential for targeting it as effective cancer immunotherapy.

## Material and Methods

### Cell Culture

Cell lines K562, A549, SKOV3, HEK293, and Phoenix AMPHO were used. Phoenix AMPHO, A549, HEK293, and SKOV3 were maintained in Dulbecco’s Modification of Eagle’s Medium (DMEM) (Corning Cellgro) with 4.5g/L glucose, L-glutamine, sodium pyruvate, supplemented with 10% heat-inactivated FBS (Atlas Biologicals). K562 cells were maintained in RPMI 1640 Medium (ThermoFisher) supplemented with 10% heat-inactivated FBS. All cell types were maintained at 37°C in humidified 95% air and 5% CO_2_ atmosphere. All cell lines were mycoplasma negative. Primary PBMC and NK cells, isolated from healthy donors, were purchased from the Human Immunology Core at the University of Pennsylvania. All primary cells were collected under a protocol approved by a University Institutional Review Board, and written informed consent was obtained from each healthy, normal donor.

## Mouse Immunization, Hybridoma Generation, and DNA-Encoded mAb Generation

Female BALB/c mice (5-7 weeks of age) were purchased from the Jackson Laboratory. Animals were housed under a pathogen-free barrier facility in accordance with NIH guidelines. All animal procedures were approved by the Institutional Animal Care and Use Committee at The Wistar Institute (protocol #112763). The mice were immunized intramuscularly (hind limbs) three times at 2-week intervals (in weeks 0, 2 and 4) with 50μg (50μl in saline) of plasmid encoding human Siglec-9 and adjuvant was mixed 1:1 (volume/volume) with CFA (Sigma-Aldrich) or IFA (Sigma-Aldrich) in a total volume of 100μl mixed by syringe. The animals then received two booster injections at two-week intervals, the first booster containing Siglec-9 DNA of identical method and the second booster containing 50μg of purified human Siglec-9 recombinant protein. Three days after the protein boost, mice were sacrificed. Their spleens were removed and fused with SP2/0 mouse myeloma cells using the HY Hybridoma Cloning Kit according to the manufacturer’s protocol using method A (Stem Cell Technologies). Hybridomas of 1152 mAb candidates were generated and screened using the ELISA method (> 0.6 O.D. 450nm in a binding assay as described below), then top 50 hits were selected for further screening using IntelliCyt-FACS analysis as described below. After characterization and sequencing the best clones, recombinant antibodies heavy and light chain sequences were assembled into the human IgG1 framework and cloned into pcDNA3.4 antibody expression vectors, as previously described (32). Plasmids were then transfected into Expi293F cells using the Expifectamine 293 Expression Kit (Thermo Fisher Scientific), and recombinant Abs have purified with protein A agarose (Invitrogen) (32).

## Enzyme-Linked Immunosorbent Assay for Hybridomas Screening

ELISA was carried out using 96-well MaxiSorp plates (Nunc) (Thermo Fisher Scientific) coated with 1µg/mL of recombinant hSiglec-9, hSiglec 7, or hSiglec 3 proteins (R&D Systems) in PBS and incubated overnight at 4°C. For the binding characterization ELISA, anti-Siglec 3 (Mouse IgG1 Clone # 6C5/2), anti-Siglec 7 (Mouse IgG2B Clone # 194212), and anti-Sigelc 9 (Mouse IgG2A Clone # 191240) were acquired from R& D systems and used as described below. Following incubation, plates were washed with PBS-T (PBS with 0.05% Tween 20) and blocked using PBS containing 10% FBS for 1 hour at room temperature (RT). Subsequently, the plates were washed with PBS-T and incubated with hybridoma, serially diluted in PBS with 1% FBS and 0.1% Tween 20 for 30 minutes on a shaker and 90 minutes at room temperature. After another wash, the plates were treated with goat-anti-mouse IgG H+L conjugated to Horse Radish Peroxidase (Bethyl Laboratories) at a dilution of 1:10000 for 1 hour at room temperature. After post-final wash, the plates were developed with SigmaFast OPD substrate (Sigma-Aldrich) for 5-10 min in the dark, and the reaction was stopped using 1N H_2_SO_4_. The plates were read using a Synergy2 plate reader (BioTek Instruments) at an optical density of 450nm (32).

For the avidity test, an in-house avidity assay was standardized using a commercial ELISA kit for detecting specific IgG antibodies modified to incorporate an elution step with urea to remove low-avidity antibodies from the target antigen. For the assay, 100µl of each diluted serum was added to wells of polystyrene plates coated with human Siglec-9 protein. All serum samples were run twice in duplicate, as described before (32).

## Production of Siglec-9-GFP Overexpressing K562 Stable Cell Lines

Human Siglec-9 was encoded in DNA using synthetic oligonucleotides. The final sequence was cloned into a mammalian expression vector (pBMN-I-GFP) followed by subsequent large-scale production (Genscript, Piscataway, NJ). Phoenix AMPHO cells cultured in a T-182 flask (Fisher) were allowed to attain 60-80% confluency and transfected with DNA using GeneJammer Transfection Reagent (Agilent Technologies) as per manufacturer’s instructions. To the transfection mixture consisting of serum-free DMEM and GeneJammer reagent (Agilent Technologies), 10μg of the plasmid (pBMN-I-GFP-hSiglec-9) was added and incubated for 30 minutes at room temperature. The transfection mixture was then added to cultured Phoenix AMPHO cells and incubated for 24-72 hours at standard growth conditions. Successful transfection was confirmed using fluorescence microscopy for the expression of GFP. The culture media-rich with lentivirus was collected 72 hours following transfection and stored at -80°C for further use (33). For cell transduction, six-well plates were coated with 10µg/mL of RetroNectin reagent (Takara Bio) and incubated overnight at 4°C. The coated plates were washed with PBS-T and blocked for 2 hours using PBS with 10% FBS at RT. Following another wash, 1mL of generated lentivirus was added to the coated wells and centrifuged for 120 min at 2000g. The supernatant from the wells was discarded, and 1 million K562 cells were added to the wells. The plates were again centrifuged for 10 minutes at 1500rpm and incubated at standard growth conditions. Successful transduction into Sigle-9-GFP overexpressing K562 cells was confirmed using fluorescence microscopy for GFP expression; cells were pooled and cultured at appropriate growth conditions for further analysis (33).

## Hybridoma Screening Using Intellicyte FACS Analysis

1x10^5^ of Siglec-9-GFP-overexpressing K562 cells co-cultured with an equal number of K562 cells in 96 healthy U-bottom plates (Fisher brand). These cells were probed with antibodies (Siglec-9 hybridoma supernatants) from clones designated positive from screening ELISA. Rat-anti-mouse IgG antibody conjugated with APC (BioLegend) at 1:200 dilution was added and incubated for 30 min at 4°C. Following incubation and washing with PBS and 1% FBS, cells were analyzed using the Intellicyt iQue screener PLUS system to identify double-positive cells (GFP and APC). Cells and beads were gated on a dot plot of side-scattered *versus* forward-scattered light intensity (33, 34).

## Cloning and Expression of a Vector System for Recombinant Antibody Expression in HEK293 Cells

We adopted the HEK293 mammalian cell line, grown at high densities in suspension, for the recombinant antibody expression. The antibody cloning method represents a simple two-step protocol with complete design flexibility. Sequence analysis was performed, and a recombinant anti-Siglec-9 mAb plasmid was constructed. Variable- or constant-region domains for human IgG1 designed within the pcDNA3.4 antibody expression cassette develop a large-scale antibody production for subsequent use in cell culture and animal model studies.

## Western Blot Analysis

Human recombinant Siglec-9, Siglec-3, and Siglec-7 proteins (R&D Systems) were reduced using NuPAGE Sample Reducing Agent (10x) (Thermo Fisher Scientific) and heated at 70°C for 10 min, then loaded onto sample lanes with Odyssey Protein Molecule Weight (LI-COR). The gel electrophoresis was carried out using sodium dodecyl sulfate-12% polyacrylamide gel for 50 min at 200V. Following electrophoresis, samples were transferred onto polyvinylidene fluoride (PVDF) membranes *via* an iBlot-2 system (Thermo Fisher Scientific) and blocked using Odyssey Blocking Buffer (35) (LI-COR) for 1-2 hours on a rocker. Membranes were treated with antibody culture supernatant (1:500) in OBB containing 0.1% Tween 20 overnight at 4°C. Following incubation, the membranes were washed four times at 5 min intervals with PBS-T. Subsequently, washed membranes were treated with goat anti-mouse secondary antibody (IRDye 800 CW) in OBB containing 0.1% Tween 20 and 0.01% SDS at a dilution of 1:10000 and incubated for 60 minutes in the dark on the rocker at room temperature. Following incubation, the membranes were rewashed four times and scanned using Odyssey CLx Imager (LI-COR).

## Antibody Peptide Mapping

Human Siglec-9 derived 20-mer peptides (GenScript) were coated on 96-well MaxiSorp plates (Nunc) (Thermo Fisher Scientific) at a concentration of 1µg/mL and incubated overnight at 4°C. After four washes with PBS-T, the plates were blocked using 10% FBS containing PBS for 2 hours at room temperature. 100µL of anti-Siglec 9 or anti-Sigelc 9 (Mouse IgG2A Clone # 191240) at 1µg/mL were added to the washed plates, and this setup was incubated for 2 hours at room temperature. Post incubation and washing, 100 µL of Goat-anti-mouse antibody conjugated with Horse Radish Peroxidase (Bethyl Laboratories) at a dilution of 1:10000 were added and incubated for 60 min at room temperature. After a final series of washing, plates were developed using SigmaFast OPD substrate (Sigma-Aldrich) for 15 min in the dark, and the reaction was stopped using 1N H2SO4. Finally, the plates were read using a Synergy2 plate reader (BioTek Instruments) at an optical density of 450nm. Each clone was screened in duplicate.

## Evaluation of NK Cell Degranulation

NK cells (effector) and K562 cells (target) at multiple effector: target ratios (ranging from 1:10, 1:5, 1:1, 1:0.5, and 1:0.1) were co-cultured. The number of effector cells was fixed at 2x105 cells per well. 10µg/mL of anti-Siglec-9 mAb was added to wells consisting of co-cultured cells, and plates were incubated for 20 hours at 37°C. Post incubation and wash, cells were stained for markers CD16 using APC Mouse Anti-human antibody (BD biosciences), CD56 using PE anti-human antibody (Bio Legend), and CD107a (a marker for NK cell activity derived from peripheral blood) using AF700 Mouse Anti-human CD107a antibody (BD Biosciences) and analyzed by Flow Cytometry using LSRII flow cytometer (BD Biosciences) (36). The analysis was done using FlowJo software (Tree Star).

## Evaluation of PBMCs Cytotoxicity Using Lactate Dehydrogenase Measurement

The cytolytic activity of human Peripheral Blood Monolayer Cells (PBMCs) against K562 targets was determined using the LDH method. The setup involved co-incubation of human PBMCs and K562 cells at an effector to target ratio of 100:1 with or without Siglec-9 mAb (1:10 dilution) for 4 hours at 37°C. Quantification of the release of endogenous LDH into the media was used as a measure for cytolytic activity. The assay was performed in triplicate, wherein PBMC+K562 is the baseline cytotoxicity with no antibody (37). The following formula was applied for calculating Cytotoxicity in this assay: % Cytotoxicity = Experimental value-Effector cell spontaneous control-Target cell spontaneous control divided by target cell maximum control-Target cell spontaneous Control.

## Detection of Cell Surface Siglec-9 Ligands

2 x 10^5^ cells were resuspended in 100µl PBS supplemented with 0.5% bovine serum albumin 0.1% sodium azide. Different amounts (beginning at 1 µg) of recombinant human Siglec-9-Fc (R&D Systems) were added and incubated for 1 h at room temperature. Following incubation, cells were washed twice and incubated with PE Fc-specific goat anti-human IgG (eBiosceince); 1:250 dilution, for 20 min at room temperature. Cells were further washed, fixed (BD Cytofix/Cytoperm), and acquired by flow cytometry (LSR II, BD). Flow cytometry data were analyzed using FlowJo software.

## Siglec-9 Ligand Detection After Desialylating Cells

5 x 105 cells were resuspended in 100 µl PBS supplemented with 0.5% bovine serum albumin 0.1% sodium azide. Sialidase (200nM or 500nM) was added and incubated for 1 h at 37°C. To remove residual sialidase, cells were washed twice by centrifugation 400g for 5 min. Next, cells were evaluated for cell surface Siglec-9 ligand content following protocol to detect cell surface siglec-9 ligands as described. Desialylated cells were also used in LDH cytotoxicity assays, as described above. Sialidase was prepared in-house using Vibrio cholerae nanH gene cloned into pCVD364 vector, which was generously provided to us by Dr. Eric R. Vimr at the University of Illinois Urbana (38).

## Ovarian Cancer Tumor Challenge in NSG and Hu-Mice Mice

For our *in vivo* examination of the potency of our blocking antibodies, we used a recently developed advanced humanized mice (hu-mice) model (30). This model harbors functional human immune cells that respond to tumor challenges (30). Immunodeficient NSG (NOD.Cg-Prkdcscid IL2rgtm1 Wjl/SzJ) mice were used to generate hu-mice by injecting CD34^+^ hematopoietic cells (i.v.) and autologous thymus (placed under renal capsule) and sequential delivery of cytokines as described before (30). Hu-mice (n=5) were then injected subcutaneously with 5x10^5^ cells of SKOV3 on day 0. Anti-human Siglec-9 (100µg) or control IgG was administered to each mouse on days 7 and 14. Tumor dimensions were measured by manual caliper on days 3, 12, 19, 24, and 33 following tumor injection. Tumor volume was calculated using the formula [tumor volume = ½ (length x width2)] (39). All mice were maintained inappropriate environmental conditions. All animal protocols were approved by Wistar Institutional Animal Care and Use Committee.

## Statistical Analysis

Differences between the means of experimental groups were calculated using two-tailed unpaired t-tests. Error bars represent the standard error of the mean. Survival rates were compared using the log-rank test. All statistical analyses were done using Graph Pad Prism 8. p < 0.05 was considered statistically significant.

## Data Availability Statement

The raw data supporting the conclusions of this article will be made available by the authors, without undue reservation.

## Ethics Statement

All animal procedures were approved by the Institutional Animal Care and Use Committee at The Wistar Institute (protocol #112763).

## Author Contributions

MA-M and KM conceived and designed the experiments for this study. HC, MH, LG, AK, OA, and AP performed the experiments. MA-M and KM wrote the original concept and manuscript, and HC, MH, and DB reviewed and edited it. MA-M and KM acquired funding; APP provided resources. MA-M and KM provided supervision. All authors contributed to the article and approved the submitted version.

## Funding

This work was part of a Wistar-Sponsored Research Agreement entitled “Targeting Siglec-9/Mucin Interaction for Cancer Immunotherapy” to MA-M and KM. Support for Shared Resources utilized in this study was provided by Cancer Center Support Grant (CCSG) P30CA010815 to The Wistar Institute.

## Conflict of Interest

MA-M and KM are named inventors of the 206193-0040-P1US patent application titled “Monoclonal Antibody Against Human Siglec-9 and Use for Cancer Immunotherapy”.

The remaining authors declare that the research was conducted in the absence of any commercial or financial relationships that could be construed as a potential conflict of interest.

## Publisher’s Note

All claims expressed in this article are solely those of the authors and do not necessarily represent those of their affiliated organizations, or those of the publisher, the editors and the reviewers. Any product that may be evaluated in this article, or claim that may be made by its manufacturer, is not guaranteed or endorsed by the publisher.
